# “I Live in a Doula Desert”: A Community-Engaged Study of Doula Care in Rural Georgia

**DOI:** 10.1089/whr.2025.0050

**Published:** 2025-06-04

**Authors:** Sydney Comstock, Alyssa Lindsey, Subasri Narasimhan, Ileana López-Martinez, Fowzio Jama, Whitney Williams, Elizabeth A. Mosley

**Affiliations:** ^1^ Center for Reproductive Health Research in the Southeast (RISE), Emory University Rollins School of Public Health, Atlanta, Georgia, USA.; ^2^ Healthy Mothers, Healthy Babies Coalition of Georgia, Atlanta, Georgia, USA.; ^3^ Converge Toward Sexual and Reproductive Health Equity, University of Pittsburgh School of Medicine, Pittsburgh, Pennsylvania, USA.

**Keywords:** community-engaged research, doula care, maternal health equity, public health, rural disparities

## Abstract

**Background:**

Maternal morbidity and mortality in the United States—and disparities therein—are significant and growing public health crises. Doulas (nonclinical perinatal support professionals) could be part of the solution, but there are significant barriers to health care in rural areas.

**Methods:**

This study examines the facilitators and barriers rural communities face in accessing doulas and ideas for expanding doula work in rural communities. As part of a larger-mixed methods, community-engaged project on full-spectrum doula care in Georgia, this study surveyed and interviewed doulas in Georgia from June 2022 to January 2023. We conducted descriptive statistics and thematically analyzed the transcripts using memoing, coding, and group discussion.

**Results:**

We surveyed and interviewed 22 doulas, 17 of whom serve rural clients including 7 who reported over 20% of their clientele are rural. Our main findings included (1) significant perinatal and social service gaps in rural areas, (2) rural poverty that impedes perinatal options, including doula access, (3) long distances between doulas, rural clients, and health care, and (4) childbirth education disparities, resulting in knowledge gaps that doulas could fill.

**Discussion:**

These results are relevant to national maternal health equity efforts in rural communities and can inform policies, programs, and future research including Medicaid reimbursement, doula training, and community-engaged research with doulas.

## Introduction

Maternal morbidity and mortality in the United States—and the disparities therein—represent a significant and growing public health crisis.^1^ Individuals giving birth in the U.S. face more dangerous health outcomes compared to those in other high-income countries.^2^ The U.S. maternal mortality ratio is 32.9 deaths per 100,000 live births,^3^ compared with 13.4 in the United Kingdom.^4^ Black Americans experience 2.6 times the risk of White Americans: 69.9 versus 26.6 per 100,000 live births.^3^ The state of Georgia has been consistently one of the worst-performing states in maternal health outcomes,^5^ with a maternal mortality ratio of 22.7 deaths per 100,000 live births.^6^ Black women in Georgia face a relatively higher maternal mortality ratio of 48.6 deaths per 100,000 live births compared with non-Hispanic White women with 22.7 deaths per 100,000 live births.^6^ There is also higher severe maternal morbidity for rural areas in the United States^7^—which the Census defines as low-density areas with populations less than 5,000—compared with high-density urban areas (populations of 50,000 or more) and moderate-density suburban areas (*i.e.,* “urban clusters” of at least 5,000 but less than 50,000).^8^ Some studies suggest that women living in Georgia’s rural counties are up to 50% more likely to experience maternal mortality compared with women in Georgia’s urban counties.^9^ Moreover, Georgia’s rural regions present additional barriers for pregnant people, such as care shortages, hospital closures, and fewer obstetric providers.^10–13^

In the face of these rural disparities and the obstacles to perinatal support, doulas could be part of the solution. Doulas are “trained professional(s) who provide continuous physical, emotional, and informational support to their client before, during, and shortly after childbirth.”^14^ Strong evidence has shown that doula care can improve maternal health outcomes, including lower risk of nonindicated cesarean delivery, more positive patient-rated childbirth experiences, lower risk of preterm birth and low birthweight, and greater success with breastfeeding.^15–22^ As a result, doula care significantly reduces health care costs.^19,20,23^ Regarding racial disparities, doula care can improve poor birth outcomes by providing accurate culturally-tailored care as well as supporting clients during their pregnancy to identify and mitigate potentially harmful mistreatment and discrimination.^24,25^ Despite the potential benefits that doula care can provide, doulas face challenges in providing their services and ensuring they are accessible to the clients they wish to serve.^26,27^

While some research has explored avenues for doula access in marginalized communities, including Medicaid reimbursement, very few have conceptualized access for those in rural geographies.^20,28,29^ In 2023, the Healthy Mothers, Healthy Babies Coalition of Georgia conducted a Medicaid Doula Pilot that successfully tested doula reimbursement and allowed for greater access to doula care for Georgia Medicaid recipients.^30^ While state Medicaid programs can address concerns about maternity care costs and quality, rural communities face care shortages, hospital closures, and fewer obstetric providers.^10^ In a 2024 report by March of Dimes, 42% of Georgia’s counties are considered maternity care deserts, compared with about 35% in the United States.^31^ There is a dearth of evidence about the expansion of doula services into rural areas, and how that could mitigate maternal and infant morbidity.

This article examines doula care in rural Georgia communities and presents potential solutions to increase access to doula care with the aim of improving pregnancy experiences and reducing rural maternal health inequities. Our team’s research questions were:

- What services and benefits are provided by doulas serving rural communities?
- What are the barriers and facilitators of doula care for rural Georgia communities?
- What solutions are possible for expanding doula care into rural communities?

## Materials and Methods

This current investigation is part of the larger Georgia Doula Study, a community-based, mixed-methods project co-led by an academic researcher—who is also a full spectrum doula from rural Georgia—and the Healthy Mothers, Healthy Babies Coalition of Georgia, a community-based, statewide nonprofit maternal and child health organization. The study was reviewed by the Emory University Institutional Review Board and was deemed exempt from oversight due to minimal risk.

To participate in our survey and in-depth interview, interested parties needed to be over 18 years old, self-identify as a doula, have worked in Georgia for at least 6 months, and speak and understand English. Recruitment, data collection, and analysis occurred between June 2022 and January 2023. Two Master of Public Health graduate student researchers (SC, IL-M) recruited all participants, conducted the interviews, analyzed the data, and discussed findings with the community partner organization. Participants were recruited through a public website with contact information (DoulaMatch.net) and through the community partner organization using their Medicaid Doula Pilot contact list, an annual conference, and a website, which includes a database of Georgia-based doulas. Snowball sampling was also used, as doulas were encouraged to share the study with doula colleagues, who could contact our team. Participants did not have a relationship with SC or IL-M before the study. All participants were informed about the purpose of the research and verbally consented. *A priori*, our team anticipated needing 20 in-depth interviews to reach thematic saturation but planned to continue until saturation was achieved.^28^ Survey data were meant to be descriptive and did not need a power calculation.

Quantitative survey data were collected using Qualtrics. The survey included the following:

- demographics
- length of time working as a doula
- training and certifications completed
- list of services they provide
- current pricing and ideal pricing
- interest in Medicaid reimbursement and community health worker classification
- demographics of their clientele, including whether they serve rural clients and what percentage of their clients live in rural areas.

Rurality was self-determined by the doula participant and operationalized as a three-level variable: “Estimate the breakdown (in percentage) of your clients by rural, urban, and suburban areas.”

The semistructured, in-depth interviews took place over Zoom and lasted about 45 minutes to 1 hour, with each conversation being audio recorded and securely stored on an encrypted server. The interview guide, which had been pilot-tested, included the following sections:

- stories about training and certification
- stories about doula services provided and payment methods
- differences between rural and urban/suburban doula care (e.g., training, certification, services, distance to care, and challenges)
- suggestions for how to improve rural doula care in Georgia

The interviews were sent to a private company for confidential transcription; transcripts were not returned to participants for review.

The survey data were cleaned and analyzed using Stata v. 14.^32^ Descriptive statistics were analyzed for all variables. Qualitative analysis was conducted using Dedoose for coding.^33^ The codebook was adapted from the previous iteration of the Georgia Doula study, with additional deductive codes related to rural care. A summary memo was created for each interview, reflecting on the research questions, positionality of the researcher, and additional inductive codes not included in the original codebook. Themes were developed through code memoing, coding matrices (*i.e.,* coded excerpts stratified by rural or urban doula care), and group discussion including the community-based organization partner.

## Results

### Quantitative survey

We interviewed 22 doulas: 17 reported serving at least some rural individuals, and seven of those reported that 20% or more of their clientele live in rural areas. Participants were diverse across race/ethnicity, socioeconomic background, doula services, and clientele served (Table 1). Most participants identified as Black or African American (36.4%) or White (40.9%). Nearly all identified as a female/woman (90.9%), one identified as nonbinary (4.6%), and one identified as both female/woman and nonbinary (4.6%). Most participants were 25–35 years of age (54.6%) or 36–45 years (31.8%). About half of the participants graduated college (54.6%), and half were employed full-time (50.0%). When asked about their scope of work as a doula, participants identified as birth/labor (86.4%), prenatal (63.6%), postpartum (54.6%), and full spectrum or abortion (22.7%) doulas. The participants charge an average of $945 for birth doula services but feel they should be paid an average of $1,279.

**Table 1.** Demographics of the Doula Sample (*n* = 22)

Variable	Frequency	Percent
Race/Ethnicity		
Black or African American	8	36.4%
White	9	40.9%
Hispanic or Latinx	1	4.5%
Biracial or multiracial	2	9.1%
Asian or Pacific Islander	1	4.5%
Other	1	4.5%
Gender Identity		
Female/woman	20	90.9%
Nonbinary or genderqueer	1	4.6%
Female/woman and nonbinary	1	4.6%
Age		
Under 25	1	4.6%
25–35	12	54.6%
36–45	7	31.8%
46–55	1	4.6%
Over 55	1	4.6%
Education		
High school	1	4.6%
Some college/technical degree	4	18.2%
Graduated college	12	54.6%
Clinical professional degree	2	9.1%
Graduate degree	2	9.1%
Missing	1	4.6%
Employment		
Yes, full-time	11	50.0%
Yes, part-time	6	27.3%
No, not looking for employment	5	22.7%
Doula Scope of Work^a^		
Prenatal	14	63.6%
Birth/labor	19	86.4%
Postpartum	12	54.6%
Full spectrum or abortion	5	22.7%

^a^ Doula Scope of Work was “check all that apply.”

### Qualitative interviews

#### Insufficient perinatal support and social service gaps

Multiple participants mentioned that rural families have little or no access to mental health, economic, and other perinatal resources including doula care. Our participants repeatedly emphasized that there is a limited number of doulas in rural areas. Amber,^a^ a White doula over 55 years old who lives in rural Georgia, said *“I live in a doula desert.”* Doulas explained, however, that they can be a protective factor in helping rural individuals connect to available services. Amelia, a White doula aged 36–45 years, explained,

“*… at any point in their pregnancy, but mostly postpartum, there’s a lack of resources locally. They usually have to travel more significantly to get to those services…lots of the services within this area are mobile and can come to them…I think there’s enough demand for it but it’s hard.”*

Amelia highlights an essential potential stopgap solution—mobile clinics—but emphasizes that reliable services for any peripartum or postpartum needs should be accessible in a timely manner. Postpartum doulas in rural areas are connecting individuals to the limited services available and providing help in the transition to parenthood.

A few doulas discussed the closing of rural hospitals, which exacerbated access to services. Amelia continued by saying,

*“We had one facility closed…every hospital is regularly at capacity here…Even recently, two of the hospitals this year I’ve had to go on diversion. For one of them, it was the first time in something like 20 years… They’re converting triage rooms to [labor and delivery] rooms.”*

Diversion occurs when a hospital is close to capacity, and ambulances are instructed to take people to other health care facilities further away.^34^

Amber explained that rural hospitals were closing and impacting their clientele even before the COVID-19 pandemic, which further strained medical services. She described the severity of perinatal service gaps, including hospital closures forcing Georgia patients to travel long distances to larger cities and even to cross state lines into northern Florida for care. She said,

*“We’ve had some rural hospitals that have closed even before COVID… but the closest hospitals are in Savannah and well, the one in [town], which is a small hospital, and beyond that Jacksonville.”*

#### Rural poverty impedes perinatal options including doula access

Our participants described this double bind: rural clients struggle to afford doula care, and doulas also struggle to support themselves financially, despite their passion to serve low-income rural clients. These doulas explained that doula care and birth centers are still perceived as luxury items in Georgia, especially in rural communities. To combat this, doulas offer sliding scales and pro bono work, but walking the line between affordable services and covering their costs can be impossible. Charlotte, a White doula aged 25–35 years, explained that she takes on several rural clients yearly who cannot afford care, so she is flexible with them. She said,

“*I would say every year I have a handful of clients who genuinely can’t afford my services. I charge the fee that I do so that I can take clients who can’t. I’ve done payment plans. I’ve had some people pay me $200, which is fine…”*

Multiple participants also discussed how rural clients’ socioeconomic status impedes their ability to have a range of options for their birth and perinatal care, thus limiting their autonomy. Several participants highlighted the multiply marginalized status of rural and impoverished communities of color in Georgia. Jada, a Black doula aged 25–35 years explained,

*“It’s literally either you go to the hospital, or you deliver at home. The majority of the time for home births, it’s an out-of-pocket expense because that’s not necessarily something that’s covered by insurance. Then when you’re thinking of communities of color… you’re pretty much telling a particular community that they have to go to a hospital setting…they can’t necessarily afford the home birth option.”*

Another doula, Logan (36–45 years old, Black participant) describes the alternative: people who have money and can drive somewhere to access a birth center. She said, *“If folks had money, they may be like, ‘Oh, I’ll go to the birthing center in Savannah or I’ll go to the [center] in Jacksonville.’”*

#### Far distances between doulas, rural clients, and health care

Multiple participants discussed how they travel far distances to serve rural clients and how their rural clientele travel long distances to access health care, including prenatal care and birth services. Maternity care deserts and the sparse population of rural health care clinics and hospitals contribute to this. Mia, a Black doula aged 25–35, told a story about one of her rural clients:

“*[She] almost didn’t even make it to the hospital… she had the baby in the wheelchair… ‘Wow, five minutes later, you would’ve had the baby in this car’…these clients are ones that I had to go to the hospital with and it was just a bit far, cell service has not been great… Before I even take a client on, I make sure I check to see the distance because again, some people labor longer, but birth is unpredictable. So, I try not to play that game…”*.

Many doulas support their clients during hospital births, but the long distances from doulas to rural clients and hospitals can limit the frequency and duration of a doula’s time with her client. This is of particular concern, because a critical component of birth doula care is continuous support. However, doulas must logistically factor in the distance when taking on clients, which further reduces the available rural doula workforce. Mia explained,

*“[It’s] a challenge, not having as much access to a hospital or facility. If you’re in an urban area, you can visit more every 5, 15, at least 20 minutes away. These ones can go 45 minutes to an hour…that has just been my challenge and my worry, whenever I think about having to service clients in those kinds of [rural] areas”*.

Kendra, a Black doula aged 25–35, similarly described,

“*In rural spaces, [the birth] is probably going to be [a] hospital birth, but the issue with those comes commuting. They have to commute to the next biggest city with the hospital unit that has the OB/GYN.”*

Another participant discussed their safety concerns about driving long distances to support clients in rural areas. Hannah, a White doula under 24 years old, explained,

“*I don’t like to do more than an hour [commute] because…if there’s bad weather then it starts to take a long time for me to get there. Then, also for my own safety, when you’re at a long birth and you’re driving home, you don’t want to be driving home for too long. Whenever I’ve done 45 minutes to an hour drive, that is enough for me to be trying to blast music and stay awake just to get [home].”*

While doulas emphasized the distance to hospital-based birth services, some doulas also discussed the distances to prenatal care that rural communities face. Jada shared,

*“[My rural client] was literally driving an hour and a half to appointments and as you get further along the appointments get closer together. Once a month, in the beginning, wasn’t so bad, but when she got down to every other week, once a week as she got closer to delivery, that becomes stressful.”*

#### Rural childbirth education disparities resulting in knowledge gaps

Many participants indicated that rural communities are unaware of doula care as an option. Even health care providers may not understand the role and scope of a doula’s work, which can lead to conflict. Mia explained,

*“I feel like, in the rural areas, its people are still trying to…accept doulas and understand the role that they play in sense as far as the medical team. I think people are still trying to adapt and adjust to that when it comes to rural areas.”*

Doulas like Mia also described how as they work with more rural clients, those clients spread their experiences through word of mouth, which is the primary way doulas find work in rural communities. For example, some clients who experience doula care benefits might recommend the service to their friends and family. However, doulas explained hospital staff still need to be educated about the role that doulas can play and the benefits they can have for patients.

Participants also described poor access to childbirth education in rural areas, which contributes to inadequate knowledge about birthing options; however, doulas are trying to help fill that gap. A few participants described how they included childbirth education in their services, whether informal (where they just explained information to their clients during visits) or formalized childbirth classes they led. One of the primary roles of a doula is as an educator, who helps their clients know what to expect, and many doulas require their clients to attend childbirth classes. Amber explained that she added childbirth education to her doula practice because,

*“[I was] less and less happy with the way I saw people being coerced, I felt, into things that were not necessarily needed. After a while, I became a childbirth educator because I saw a need for people to have more education about what was going on and what to expect, and what they could and couldn’t do.”*

Participants discussed how a lack of education in rural communities contributes to clients not knowing they can advocate and ask for different options during birth. However, it is unclear if birthing clients can demand services not yet available in their area. Charlotte shared,

*“I think the education gap is huge. I think there’s just a lack of options here. I’m trying to figure out why that is…no hospitals here offer waterbirth, so no one knows to ask about it.”*

Another primary role of doulas is to support person-centered birth plans. However, the combined lack of knowledge about perinatal care possibilities (including the work of doulas) and the structural lack of birthing options restricts clients’ full autonomy.

## Discussion

The findings from this community-engaged study showcase which doula services are currently offered to rural communities in Georgia as well as existing rural perinatal health care and social service gaps. Our results increase the understanding of rural doula services and their potential benefits in Georgia and, by extension, other rural communities across the United States. Primary barriers to doula care include lack of childbirth education, low knowledge about and awareness of doulas, limited access to childbirth options, long distances to perinatal care and support, and rural poverty that makes out-of-pocket doula costs impossible for many clients, while also limiting self-employed doulas from providing care to communities with the greatest need. Despite these challenges, doulas focus on creative solutions including sliding scale fees, pro bono services, and the provision of childbirth education for their clients. Standard doula training includes skills-building as basic childbirth educators,^35,36^ which serves as a facilitator to meet these needs of rural clients. Rural-serving doulas have recognized that their clients need access to childbirth education, including increased awareness about birthing options, and are stepping into that gap. Rural-serving doulas could particularly benefit from cross-training in childbirth education.

In many ways, doulas are working to address and mitigate these negative social determinants of health including limited access to childbirth education, birthing options, and social services. Researchers have previously documented the important role of doulas in addressing social determinants of health to facilitate “good birth” experiences.^24^ We have extended this existing research by demonstrating how doulas function in this capacity for rural communities. The health care and social service barriers rural communities face in Georgia are similar to other states with large rural populations and major urban−rural inequities.^37–39^ Rurality affects the health and social services available, with individuals often encountering barriers such long distances to care with limited transportation options, workforce shortages, gaps in insurance coverage, poor broadband access, lower health literacy, greater social stigma, and concerns for privacy—all of which make it difficult to obtain appointments and reach services.^38^ Prenatal care, childbirth education, labor and delivery services, and postpartum support are essential for healthy pregnancies, therefore barriers to this care contribute to greater burden of maternal morbidity and mortality.^9^

Long distances to health care services is a ubiquitous issue for rural communities in Georgia and across the United States,^10,11,38,39^ and this is no different for doula care. The doulas in our study, and who are listed in existing doula registries, are most likely to live in urban areas and drive out to clients living in rural areas. This underscores the need to identify, train, and mentor new doulas, who live in rural communities, and to support existing rural-dwelling doulas to be identified by state and national doula registries. Rural doulas would be particularly well-positioned to offer culturally-tailored perinatal support and childbirth education and to connect rural clients with health care and social services.

Widespread rural poverty^38^ is a significant barrier for pregnant people needing to access and pay for doula services. While doulas try to offer sliding scale fees and, at times, provide pro bono services, this negatively impacts their own financial security. The tension between doulas wanting to serve low-income clients, while also needing to make a living has been previously documented by our Georgia Doula Study^26^ and other studies focused on Medicaid doula services.^20,28–30^ Based on this evidence, one recommendation is for Medicaid to cover doula care and reimburse at rates high enough for doulas to make a living wage and to cover the costs of travel when needed.^29,40,41^ By covering doula care for pregnant people living on low incomes, doulas will be able to serve those communities sustainably, and their rural clients—who are at higher risk of poor maternal and child health outcomes—will have access to health-protective care. Studies consistently show that Medicaid funding for doula care ultimately reduces health care costs by averting Cesarean delivery and poor birth outcomes.^23,28^

Additional policy and practice recommendations stemming from this study and identified by prior research include certifying rural doulas as perinatal community health workers,^42^ increasing access to virtual doula services (which necessitates widespread investment in rural broadband),^43^ and building trust and relationships between doulas and health care providers including hospitals.^44^ In fact, Healthy Mothers, Healthy Babies Coalition is currently leading a Doula Integration Pilot at two hospitals in Georgia, including one in a rural county in North Georgia.^45^

## Conclusions

Findings from this study carry important implications for public health and perinatal practice, research, and policy. Doulas are doing their best to provide perinatal support services and childbirth education to clients in rural areas of Georgia, despite numerous barriers. Doulas are uniquely well-situated and trained to provide education, advocacy, empowerment, and continuous support that is critical for improving rural maternal and infant health outcomes. Doulas are already working across the United States with rural-dwelling pregnant people, which alleviates burden on health professionals and enhances patient experiences and outcomes. Rural maternal and child health indicators can be improved by making doula care accessible and affordable for rural communities. Research has repeatedly illustrated that doulas benefit maternal and child health and reduce health care costs;^15,16,23,28,29^ now, the doula workforce needs to be scaled up and financed, especially in rural areas. Public health and perinatal organizations and leaders must advocate for implementing evidence-based policies—including doula Medicaid coverage—to maximize doula benefits and to reduce rural−urban perinatal disparities.

## Notes

^a^
Pseudonyms are used throughout.
